# Dual targeting of PD-1/PD-L1 and iL-33/ST2 signalling pathways: a promising approach in breast cancer immunotherapy

**DOI:** 10.1080/07853890.2025.2593198

**Published:** 2025-11-24

**Authors:** Milan Jovanovic, Nevena Gajovic, Miodrag Jocic, Marina Jovanovic, Milena Jurisevic, Ivan Jovanovic

**Affiliations:** a Department of Abdominal surgery, Military Medical Academy, Belgrade, Serbia; b Medical Faculty of the Military Medical, Academy University of Defense, Belgrade, Serbia; c Center for Molecular Medicine and Stem Cell Research, University of Kragujevac, Kragujevac, Serbia; d Institute for Transfusiology and Haemobiology, Military Medical Academy, Belgrade, Serbia; e Department of Otorhinolaryngology, University of Kragujevac, Kragujevac, Serbia; f Department of Pharmacy, University of Kragujevac, Kragujevac, Serbia; g Department of Medicine, University of East Sarajevo, Foca, Bosnia and Herzegovina

**Keywords:** Breast cancer, PD-1/PD-L1 pathway, IL-33/ST2 pathway, immunotherapy, combination therapy

## Abstract

**Introduction:**

The immune checkpoint axis PD-1/PD-L1 is a cornerstone of cancer immunotherapy. However, its efficacy in breast cancer is often limited by a complex and immunosuppressive tumour microenvironment (TME). The IL-33/ST2 signalling pathway, a key player in type 2 inflammation, is increasingly recognized for its pro-tumoral roles in various cancers, contributing to immune evasion and metastasis. This review explores the synergistic potential of combining PD-1/PD-L1 blockade with IL-33/ST2 inhibition to overcome therapeutic resistance and enhance anti-tumour immunity in breast cancer.

**Methods:**

Current literature on the PD-1/PD-L1 and IL-33/ST2 pathways in breast cancer progression was synthesized, focusing on their mechanisms of immune suppression and TME modulation. It examines preclinical and clinical data on the individual and combined therapeutic strategies targeting these pathways.

**Results:**

Evidence suggests that IL-33 signalling promotes a suppressive TME by recruiting regulatory T cells, myeloid-derived suppressor cells, and M2 macrophages, which can limit the effectiveness of PD-1/PD-L1 inhibitors. Conversely, blocking the IL-33/ST2 pathway has been shown to reprogram the TME, leading to increased infiltration of cytotoxic T lymphocytes and enhanced anti-tumour responses. Therefore, a dual-targeting approach is proposed to simultaneously disarm these two distinct but cooperative immune evasion mechanisms.

**Conclusion:**

Dual blockade of PD-1/PD-L1 and IL-33/ST2 signalling pathways represents a novel and promising strategy to enhance the efficacy of immunotherapy in breast cancer. This approach has the potential to revert the immunosuppressive TME, leading to more durable and robust anti-tumour responses. Further research is warranted to validate this hypothesis and translate it into effective clinical trials.

## KEY MESSAGES

- While immune checkpoint inhibitors targeting the PD-1/PD-L1 axis have shown clinical efficacy in triple-negative breast cancer, variable therapeutic responses highlight the need for additional targets and more precise biomarkers.
- The IL-33/ST2 signalling pathway plays a complex, dual role in the tumour microenvironment, acting as both a pro-tumorigenic and an anti-tumorigenic regulator of immune responses depending on the context.
- Combined immunotherapeutic strategies that simultaneously modulate both the PD-1/PD-L1 and IL-33/ST2 pathways show a synergistic potential to enhance anti-tumour immunity and overcome resistance in breast cancer.

## Introduction

Breast cancer remains the most frequently diagnosed malignant tumour and the leading cause of cancer-related death in women globally, with 2.3 million new cases and 666,000 deaths reported in 2022, highlighting its persistent global health challenge [1–3]. Despite advancements in screening and therapeutic approaches, breast cancer’s complex, multifactorial aetiology and heterogeneous nature present significant clinical challenges, including unpredictable responses, recurrence, and metastasis. Understanding contributing factors like genetic predisposition, environmental, and lifestyle elements is crucial for improved prevention and early detection [4]. Beside tumorigenesis, another important step in malignant disease is tumour progression that depends also on several intrinsic factors such as characteristics and malignant potential of tumour cell and extrinsic factors such as tumour microenvironment, immune system etc. [5].

Breast cancers are primarily classified into invasive carcinoma and lobular types [6]. Further classification of breast cancers in four main types luminal A, luminal B, HER2-positive, and Triple-negative (TNBC) subtypes is based on histological grade, oestrogen/progesterone receptor, HER2, and Ki67 expression [7]. TNBC, lacking ER, PR, and HER2, presents the most challenging therapeutic and prognostic outlook, as it is unsuitable for endocrine or HER2-targeted therapies [8]. TNBC is an aggressive cancer subtype associated with increased distant metastasis, recurrence, poor prognosis, and shortened overall survival [8,9]. Frequent genetic alterations in TNBC include p53, PIK3CA, BRCA1, BRCA2, and ATM mutations [10,11]. Their critical role in DNA-damage response pathways highlights their significance in tumorigenesis.

Current breast cancer treatments include surgery, chemotherapy, radiotherapy, endocrine, targeted, and immunotherapy. While surgery and chemotherapy can cure non-metastatic disease [12], systemic treatment remains primary for metastatic forms. Endocrine therapy is standard for hormone receptor-positive tumours, with response correlating to ER/PR expression [13]. The development of anti-HER2 antibodies has significantly improved outcomes for HER2-positive patients, with combination therapy now standard [14]. Beside all of these standard therapy modalities, the greatest challenge is the treatment of TNBC. As triple negative type of tumour, chemotherapy is the primary modality, yet the ‘TNBC paradox’ highlights initial responsiveness followed by high recurrence and lethal outcomes due to its aggressive nature and lack of efficient therapies [15]. Epidemiological data show less than one-third of women with metastatic breast cancer survive 5 years, with metastatic TNBC universally fatal [16].

Platinum-based therapy is significant for TNBC, but is limited by frequent side effects like myelosuppression, ototoxicity, and neurological/kidney issues [17]. Furthermore, resistance often develops over time, leading to reduced responsiveness and evasion of programmed cell death [18]. Immunotherapy has slightly improved TNBC outcomes [19]. Recognizing ‘immune surveillance’ as key for eliminating mutated cells [20], research shifted from direct immune stimulation to immune checkpoint inhibitors. Confirmation that TNBC cells express significantly higher amount of PD-1 molecule or RNA specific for PD-1, classified it as ‘immunogenic’ justifies examination of immunotherapy as therapy option [21,22]. Although TNBC has been the principal focus of PD-1/PD-L1 clinical activity due to higher PD-L1 expression and TIL density, it is important to note distinct response patterns across breast cancer subtypes. HER2-positive tumours display intermediate immune infiltration and may derive benefit from checkpoint blockade particularly when combined with HER2-targeted agents, while luminal (ER+/PR+) tumours generally exhibit lower baseline immunogenicity and variable PD-L1 expression, often associated with poorer responses to monotherapy checkpoint inhibition [23]. Thus, subtype-tailored strategies focused on combination with subtype-specific agents are critical when designing trials of PD-1/PD-L1 + IL-33/ST2 modulation.

Beyond PD-1/PD-L1, the IL-33/ST2 pathway has gained attention in antitumor immunity [24]. The fact that its expression in tumour tissue correlates with cancer progression and prognosis shed a new light on the exploration of IL-33/ST2 signal pathway in breast cancer [24–26]. As an alarmin released from necrotic cells, IL-33 modulates antitumor immune responses and influences tumour biology, including angiogenesis and proliferation [27,28]. Moreover, as a cytokine, IL-33 can modulate immune response [29]. Its role in malignant disease progression makes IL-33/ST2 a focus in oncology and immune-oncology studies. Recent evidence indicates that epigenetic properties contribute significantly to breast cancer heterogeneity and immune regulation. Additionally, those epigenetic alterations, as a potential modulator of apoptotic and stress-response pathways, has been recognized as a multifunctional regulator implicated in tumour progression, therapeutic resistance, and immune evasion [30,31], which further point on necessity of dual-pathway targeting in diverse breast cancer populations.

This review systematizes current knowledge on the immunomodulatory effects of PD-1/PD-L1 and IL-33/ST2 signalling pathways in breast cancer antitumor immunity.

## Discussion

### The PDL/PD1 signalling pathway: role in immune response regulation, and effects on specific immune cells

The discovery of programmed death ligand 1 (PDL1) and its receptor, programmed death 1 (PD1), has deepened the understanding of the modulation and suppression of the immune response, especially the antitumor immune response. PDL1 and PD1 form a critical immune checkpoint axis that modulates the balance between immune activation and immune tolerance. The PD-1 receptor, encoded by the PDCD1 gene, was first identified in 1992 during investigations into apoptosis-related genes in murine models [32]. Its essential role in immune inhibition was dissect out in the early 2000s. The discovery of its ligands PD-L1 (CD274) and PD-L2 (PDCD1LG2), and their interaction with PD-1-expressing T cells [33] has made this knowledge possible. The PDL/PD1 signalling pathway was originally considered part of the peripheral tolerance mechanism that prevents autoimmunity. It is now recognized as a one of the key regulators of both innate and adaptive immune responses [32,33]. The interaction between PDL1 or PD-L2, expressed across multiple cell types including dendritic cells (DCs), macrophages, endothelial cells, and numerous tumour cells, and PD1, which is predominantly found on activated T cells, B cells, natural killer (NK) cells, and myeloid cells, delivers a potent inhibitory signal. Activation of this signalling pathway reduces T cell receptor (TCR) signalling and limits T-cell proliferation, cytokine production, and cytotoxic activity [34,35]. Subsequently, this checkpoint plays a major role in limiting excessive immune responses and maintaining homeostasis of immune system. Recent clinical and experimental studies have shown that within the tumour microenvironment (TME), cancer cells often upregulate PDL1 expression to facilitate immune evasion and tumour progression [35–37]. Tumour-infiltrating lymphocytes (TILs), chronically exposed to antigens in the TME, consistently express PD-1 and have reduced effector function-hallmarks of T cell exhaustion [36,37]. Understanding this phenomenon is crucial before delving into its implications in the tumour environment, where immunosuppression dominates. Regulatory T cells (Tregs), known for dampening immune responses, frequently express elevated PD1, and growing evidence suggests that PD1 signalling promotes Treg stability and suppressive function, thereby contributing to an even more immunosuppressive TME [37,38]. This adds another layer of complexity to anti-tumour immunity.

Additionally, myeloid-derived suppressor cells (MDSCs) and tumour-associated macrophages (TAMs) have been shown to express PD-L1, enhancing immune evasion [39]. New studies reveal that even NK cells, traditionally considered effectors of innate immunity and first-line defenders against transformed cells, can express PD1 under certain pathological conditions [40]. Within the TME, PDL/PD1 signalling engagement can significantly impair NK cell cytotoxicity, thereby expanding the spectrum of immune suppression mediated by this pathway [41]. In line with this finding, our recent study in a murine breast cancer model revealed that anti-PD1 therapy, beside enhancing T cells activity, activates NK cells, increasing expression of FasL, NKG2D, production of IFN-γ while simultaneously reducing inhibitory markers such as Foxp3 and IL-10 [42]. These effects were observed both systemically and within the tumour, suggesting that PD-1 blockade facilitates cytotoxic phenotype in both T and NK cells and reinforces the anti-tumour immune response [42]. Similarly, in our previous study, Jovanović et al. demonstrated that anti-PD-1 treatment activates NKT and dendritic cells in a same murine breast cancer model, by increasing the expression of tumoricidal molecules such as perforin and promoting M1 polarization of TAMs, leading to overall tumour growth deceleration [43].

PDL/PD1 signalling targets not only effector cells but also an antigen-presenting cells, such as DCs and macrophages. For instance, DCs can increase PDL1 expression in response to proinflammatory cytokines, subsequently inhibiting T cell priming while inducing T cell energy [44]. TAMs, particularly M2 macrofages, frequently express high levels of PDL1, facilitating the local immunosuppressive surrounding [45]. This may create a feedback loop that potentiate immune evasion.

PDL1 expression is highly dynamic and dependents of various signalling networks. Pro-inflammatory cytokines such as IFN-γ, hypoxic stress, and intrinsic oncogenic signalling pathways can increase local PDL1 expression. This phenomenon allows tumours to generate adaptive resistance against constant immune pressure [46]. This plasticity poses a challenge for sustainable immunotherapy.

In summary, the PDL/PD1 signalling pathway presents immune response regulator under physiological conditions. However, in malignancies, this signalling pathway participates in mechanisms to suppress effective antitumor immunity. Understanding the different roles of PDL1 and PD1 in various immune cell types is essential for the rational design of immune-based therapy. The discovery of immune checkpoint inhibitors targeting the PD1/PDL1 axis has transformed cancer treatment paradigms, especially in tumours characterized by high mutational burden or pre-existing immune infiltration [47]. These therapies have underscored that restoring T-cell function *via* PD1 inhibition can achieve durable responses in select patient populations. Still, considerable gaps remain in our knowledge of how this pathway interfaces with other immune circuits, emphasizing the need for continued exploration and refinement of immunotherapeutic strategies [42,43].

### The PDL/PD1 signalling pathway in breast cancer

Breast cancer has historically been considered less immunogenic in comparison to melanoma or non-small cell lung cancer. Today, a growing body of evidence points to the immunogenicity of breast tumours and the role of antitumor immunity in the biology of these tumours, highlighting the importance of immune evasion mechanisms, including the PDL-1/PD1 axis [48,49]. This signalling pathway has come into focus in breast cancer research due to its impact on tumour behaviour and its role in modulating antitumor immunity and immune evasion. Although initially studied only in highly immunogenic malignancies, it is now being investigated and used in many malignancies. Studies now revealed that certain types of breast cancer can exploit the PDL-1/PD1 pathway to evade immune surveillance [21].

It is now known that in specific subtypes of breast cancer, especially triple-negative breast cancer (TNBC) and HER2-positive tumours, increased leukocyte infiltration and PD-L1 expression have been detected. This finding implicates a prominent role for this axis in disease progression and immune evasion [46,48]. Breast cancer cells upregulate PD-L1 expression through a variety of intrinsic and extrinsic mechanisms. Oncogenic signalling pathways such as PI3K/AKT, MAPK, and MYC affect constitutive PD-L1 expression [50,51], while pro-inflammatory cytokines, particularly interferon-gamma (IFN-γ) produced by tumour-infiltrating lymphocytes (TILs), induce adaptive PD-L1 expression [52].

PD-L1 expression varies among various breast cancer molecular subtypes. In TNBC, a tumour that does not express oestrogen receptor (ER), progesterone receptor (PR), and HER2, elevated PD-L1 expression is frequently detected. This increased PD-L1 expression is often accompanied by a more active immune microenvironment in the tumour, including increased TIL density. This may predict better responses to checkpoint inhibition [53]. PD-L1 can be expressed on both tumour and tumour infiltrating immune cells and is often associated with an immunosuppressive microenvironment, characterized by the accumulation of regulatory T cells (Tregs), exhausted CD8^+^ T cells, and increased expression of additional inhibitory molecules such as CTLA-4 and TIM-3 [54]. Activation of the PDL-1/PD1 signalling pathway on effector T cells leads to suppression of cytotoxic function, facilitating tumour immune escape despite the intense immune infiltration [55].

The prognostic significance of PD-L1 expression in breast cancer is not always clear and is sometimes contradictory. Some studies have associated high PD-L1 expression with more aggressive tumours, larger size, higher grade, and hormone receptor negativity [56]. Other studies have revealed that in basal-like or TNBC tumour subtypes, overexpression of PD-L1 correlates with improved metastasis-free survival and overall survival, especially when intense leukocyte infiltration was detected [49,57]. In TNBC, PD-L1 expression, particularly on tumour-infiltrating leukocytes, often reflects more active anti-tumour immunity and is associated with better outcomes [58]. In contrast, in hormone receptor-positive tumours, which typically have a less intense anti-tumour immune response, PD-L1 expression may indicate a more immunoresistant phenotype and is associated with a worse prognosis [59]. These opposing observations highlight the dual nature of PD-L1 as a mediator and modulator of the antitumor immune response.

Targeting the PDL-1/PD1 signalling axis has redefined treatment strategies in oncology. When it comes to breast cancer patients, immune checkpoint inhibitors have shown clinical benefit, particularly in patients with PD-L1-positive TNBC. The combination of atezolizumab (anti-PD-L1 mAb) with nab-paclitaxel was the first such immunotherapy treatment approved for breast cancer, demonstrating a significant progression-free survival advantage in patients with PD-L1-positive metastatic NTBC [60]. One pivotal trial demonstrated that adding atezolizumab to nab-paclitaxel improved progression-free survival in patients whose tumours expressed PD-L1 on tumour-infiltrating leukocytes [60]. More recently, pembrolizumab (anti-PD-1 mAb) has shown promising results in both early-stage and metastatic NTBC, especially when combined with chemotherapy or other immunomodulatory agents [61].

Despite these advances in therapeutic modulation of antitumor immunity, therapeutic responses in breast cancer remain modest compared with more immunogenic tumours. A significant number of PD-L1-positive patients simply do not respond or respond poorly to therapy, and both primary and acquired resistance are common. Factors contributing to poor response include low tumour mutation burden, immunosuppressive microenvironment, and technical variability in PD-L1 testing [62]. Factors that further contribute to resistance include the absence of pre-existing antitumor immunity, compensatory expression of alternative immune checkpoints, and tumour-intrinsic factors such as activation of the β-catenin pathway or loss of PTEN [63]. In response to this phenomenon, research is increasingly focusing on combination approaches that integrate checkpoint inhibitors with chemotherapy, radiotherapy, targeted agents, cancer vaccines, or oncolytic viruses to enhance tumour immunogenicity and clinical outcomes of therapy [64].

Although the PD-1/PD-L1 signalling pathway represents a key mechanism of immune evasion in breast cancer, it also represents a promising therapeutic target. The search for a predictive biomarker of therapeutic outcome remains a critical challenge. Immunohistochemical (IHC) evaluation of PD-L1 expression is the current standard for patient selection [65]. However, its limitations, including assay variability, dynamic PD-L1 expression, highlight the need for more robust, integrative biomarkers of therapeutic outcome. Ongoing studies aim to define better predictive markers, optimize combination therapy strategies, and further elucidate the mechanisms of tumour resistance to applied therapy. Promising findings include TIL profiling, interferon gene expression profiling, and spatial analysis of immune cell localization within the tumour microenvironment [66].

### The IL-33/ST2 signalling pathway: discovery, role in immune response regulation, and effects on specific immune cells

Interleukin-33 (IL-33) is a member of the IL-1 cytokine family [67]. It functions as an alarmin that is released following cell injury or stress, thereby initiating immune responses [67]. IL-33 was first identified in 2005., as a nuclear factor in high endothelial venules of lymphatic tissue [68]. It regulates gene transcription, and acts simultaneously as a cytokine and a nuclear factor [68]. Three very potent inflammatory cytokine members of IL-1 cytokine family are IL-1α, IL-1β, IL-18. IL-33 is described like a new member of the IL-1 family that induces an immune response trough Th2 interaction the ST2 receptor complex [69]. This action of IL-33 is in contrast to the characteristics of other cytokines from the IL-1 family, such as IL-1β and IL-18, which stimulate a Th1 immune response [69].

**Figure 1.**
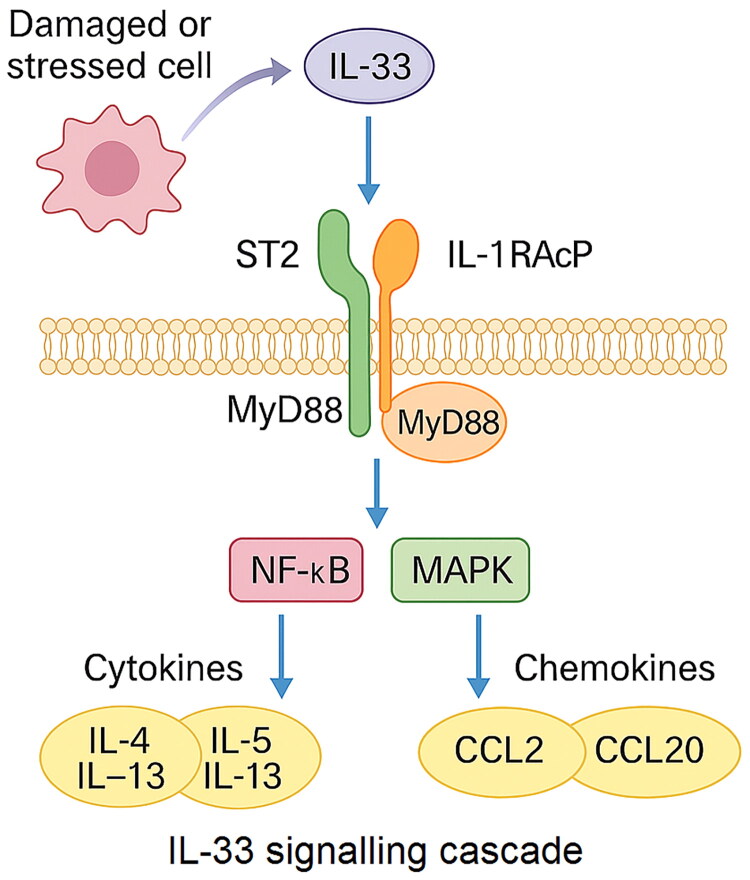
IL-33/ST2 signalling cascade. *Notes:* IL-33 is released as an alarmin from damaged or stressed epithelial and stromal cells, where it binds to the ST2/IL-1RAcP receptor complex on target immune and tumour cells. This engagement recruits the adaptor protein MyD88 and triggers downstream activation of NF-κB and MAPK signalling pathways. As a result, a broad panel of effector molecules is produced, including type 2 and type 3 cytokines (IL-4, IL-5, IL-13, IL-17), chemokines such as CCL2 and CCL20, and growth factors that orchestrate immune cell recruitment, polarization, and activation. Through these events, IL-33/ST2 signalling shapes the tumour microenvironment, promoting pro-tumorigenic inflammation but also creating opportunities for immune modulation when therapeutically targeted.

IL-33 exerts its effects by binding to the ST2 receptor (also known as IL1RL1), which exists in two major isoforms: a membrane-bound form (ST2L) and a soluble form (sST2). The ST2L is responsible for signal transduction, while sST2 acts as a decoy receptor, neutralizing IL-33 activity [69–71]. IL-33 binds to a receptor complex formed by ST2L and IL-1 receptor accessory protein (IL-1RAcP), thereby activating downstream signalling pathways such as NF-κB and MAPK. Activation of this signalling cascade leads to the production of type 2 cytokines, chemokines, and growth factors, orchestrating a variety of innate and adaptive immune responses (Figure 1) [72].

The impact of the IL-33/ST2 signalling axis on various immune cells is highly context-dependent and often dichotomous [69]. When it comes to innate immunity, IL-33 potently stimulates innate lymphoid cells type 2 (ILC2s), facilitating their proliferation and secretion of type 3 cytokines such as IL-5 and IL-13, which are essential in responses to parasitic infections and allergic inflammation [73]. IL-33 also acts on mast cells, central mediators of allergic diseases, by enhancing their degranulation and cytokine production, subsequently contributing to inflammation and tissue remodelling. Eosinophils and basophils similarly respond to IL-33, enhancing type 2 immunity and potentially facilitating fibrosis and tumour progression [74]. These effects of IL-33 are generally protective in the context of inflammation and tissue repair. However, in the tumour microenvironment, IL-33 can facilitate either pro-tumorigenic or anti-tumour responses, depending on the broader immune landscape [75]. Notably, our studies have highlighted a role of IL-33 in systemic manifestations of malignant diseases, such as inflammation-related anaemia and thrombocytosis in advanced stages of colorectal cancer, suggesting that IL-33 may modulate not only local immune responses but also systemic alterations associated with disease progression [76,77].

When it comes to acquired immunity effect, IL-33 exhibits multiple functions. It enhances the clonal expansion and effector function of Th2 cells, thus favouring type 2 immune polarization, but can also facilitate the survival and cytotoxicity of CD8^+^ T cells under certain conditions [78]. IL-33 has been shown to promote the accumulation and suppressive capacity of regulatory T cells (Treg), contributing to immune tolerance and tissue repair, as well as facilitating immune evasion in cancer [79,80]. IL-33 also affects B cells by promoting antibody synthesis, particularly in Th2 microenvironments. It modulates dendritic cells (DCs) and macrophages, enhancing their maturation and antigen presentation. However, chronic exposure to IL-33 can polarize macrophages towards M2 immunosuppressive phenotype [81].

Several studies indicate that IL-33 directly acts on ST2-expressing CD4^+^ T cells, enhancing Th2 polarization [69]. At the same time, it exerts immunoregulatory effects, enhancing the expansion and suppressive function of ST2^+^ Tregs and contributing to immune tolerance in both homeostatic and pathological conditions, including malignancy [82]. Conversely, during acute inflammation or in the setting of immunotherapy, IL-33 can stimulate effector CD8^+^ T cells and enhance their cytotoxic function, suggesting its dual immunomodulatory potential [83].

This dual nature of IL-33, enhancing inflammation while promoting immunoregulation, highlights the context-dependent nature of IL-33/ST2 signalling [84–86]. In acute injury, the release of IL-33 (as an alarmin) is usually beneficial, promoting rapid immune responses and tissue regeneration [75]. In contrast, chronic signalling within the tumour microenvironment or chronic inflammatory processes can lead to immunosuppression, fibrosis, and disease progression [75]. Short-term effects of IL-33 on macrophages and dendritic cells include activation, cytokine production, and enhanced antigen presentation, potentially enhancing antitumor immunity under favourable conditions [87]. Furthermore, IL-33 can enhance the survival and cytotoxicity of NK cells, especially when co-stimulated with IL-12 or IL-18, suggesting a role in enhancing innate antitumor responses [88]. However, long-term exposure can shift the macrophage phenotype towards a tumour-supportive M2-like phenotype [89].

The biological activity of IL-33 is finely regulated by multiple mechanisms, including the presence of sST2, proteolytic degradation, and oxidative inactivation of IL-33 itself, which together modulate its availability and function [90]. The balance between membrane-bound ST2L and soluble sST2 is especially critical in determining the outcome of IL-33/ST2 pathway activation [91]. sST2 serves as a natural buffer by sequestering and subsequently neutralizing extracellular IL-33, thereby reducing its availability and thus activity [92]. Elevated levels of sST2 are associated with poor prognosis in several malignancies, potentially due to suppressed IL-33-mediated immune responses [93]. Therefore, both the timing and amount of IL-33 release, together with the presence and ratio of its receptor isoforms, shape the outcome of the immune response.

In summary, the IL-33/ST2 signalling pathway represents a multifaceted and dynamic axis that affects a wide range of immune cell types and responses. Its effects vary depending on tissue context, cytokine milieu, and disease state, encompassing roles in inflammation, immune activation, tolerance, and tissue repair. Its dual ability to promote both active protective immunity and immunosuppression positions IL-33/ST2 axis as a therapeutic target in diseases marked by immune dysregulation, including cancer.

### The IL-33/ST2 signalling pathway in breast cancer: tumour biology, prognostic significance, and therapeutic targeting

The IL-33/ST2 signalling axis has attracted increasing interest in breast cancer research due to its diverse and sometimes contradictory effects on tumour progression and immunoregulation. IL-33 is released from necrotic or stressed stromal and epithelial cells (as an alarmin) and can significantly affects the tumour microenvironment (TME) by modulating both innate and acquired anti-tumour immune responses. However, its effects are not uniform. Under certain circumstances, IL-33 promotes tumour growth and immune evasion, while in others it can enhance antitumor immunity [80,94–96]. By using a 4T1 mouse model of breast cancer, we showed that IL-33 administration led to accelerated tumour growth and accelerated metastasis into lung and liver. This effect was accompanied by the accumulation of immunosuppressive cells such as CD11b^+^Gr-1^+^ MDSCs, ST2^+^ IL-10-producing Tregs, and type 2 innate lymphoid cells (ILC2) [80].). Furthermore, IL-33 treatment was associated with reduced accumulation and cytotoxicity of intratumoral NKp46^+^ NKG2D^+^ NK cells, but also enhanced neovascularization, all of which together highlight the pro-tumorigenic potential of the IL-33/ST2 axis *in vivo* [80].

Within the TME, IL-33 is produced by a variety of cell types, including stromal fibroblasts, endothelial cells, and occasionally tumour cells themselves. Its release (as an alarmin) is often triggered by tissue damage, hypoxia, or chemotherapy-induced stress, conditions that are frequently encountered in tumours [67,97]. Once secreted, IL-33 interacts with immune cells expressing ST2, initiating downstream signalling cascades in the same cells that can ultimately lead to tumour suppression or promotion, depending on the prevailing immune context and local microenvironment.

Microenvironmental conditions critically shape IL-33/ST2 signalling outcomes. Hypoxia and tissue damage, common in rapidly growing tumours, promote passive IL-33 release from necrotic stromal/epithelial cells and activate stromal cancer associated fibroblasts (CAFs), which can amplify IL-33 production and secrete chemokines that recruit Tregs and MDSCs [98]. These events favour an immunosuppressive, pro-tumour milieu. Conversely, in tumours with pre-existing effector infiltration (high TILs, active IFN-γ signalling), transient IL-33 release can facilitate CTLs and NKs cytotoxicity [99]. Mechanistically, these divergent outcomes are mediated through context-dependent downstream signalling (e.g. NF-κB and MAPK), differential expression of ST2 isoforms (membrane ST2L vs soluble sST2), and cellular composition of the stroma [28].

Increased expression of IL-33 and ST2 has been detected in breast cancer tissues compared with surrounding normal tissue, although the prognostic implications remain unclear. Our previous study indicated a positive correlation between advanced stage of breast cancer and increased expression of IL-33. Endogenous IL-33 mRNA level in mammary tumours showed significant time-dependent increase with the highest levels detected at day 28 after tumour challenge (fourfold increase in expression compared to the initial one) [80]. Similarly, flow cytometric analyses revealed time-dependent increase of IL-33 expression in mammary tumours during cancer progression [80]. Another study revealed that IL-33 levels and the IL-33/IL-12 ratio were significantly higher in stage IV breast cancer patients than other stages and controls (*p* < 0.0001 and *p* < 0.001, respectively) [100]. In some patient cohorts, increased IL-33 expression has been associated with higher tumour grade, lymph node metastasis, and reduced overall survival, suggesting an association with tumour promotion [24]. This effect appears to be mediated, at least in part, by the recruitment and activation of immunosuppressive cells such as M2-like tumour associated macrophages (TAMs), myeloid-derived suppressor cells (MDSCs), and ST2^+^ regulatory T cells (Tregs), all of which contribute to immune evasion and subsequent tumour progression [80,96].

In addition, IL-33/ST2 signalling seems to be involved in processes critical for metastasis, including epithelial-mesenchymal transition (EMT), extracellular matrix remodelling, and angiogenesis [97]. By enhancing the production of matrix metalloproteinases and vascular endothelial growth factor (VEGF), IL-33 creates an environment conducive to tumour invasion [97,101]. These mechanisms are particularly pronounced in aggressive subtypes such as triple-negative breast cancer (TNBC), where IL-33-rich microenvironments can drive disease progression [80,102].

In contrast, IL-33 has also been shown to stimulate potent antitumor immune response under specific microenvironmental conditions. It can enhance the cytotoxic activity of CD8^+^ T cells, natural killer (NK) cells, and dendritic cell maturation and ability to present tumour antigens, contributing to more efficient tumour surveillance [99]. ST2^+^ NK cells, in particular, display greater activity and enhanced cytotoxicity when stimulated with IL-33, a phenotype associated with prolonged progression-free survival. These effects are often enhanced in the presence of costimulatory cytokines such as IL-12 and IL-18 or when immune checkpoint pathways are pharmacologically inhibited [71]. Results from studies in experimental models of malignancy further suggest that intratumoral administration of IL-33 can promote effector T cells infiltration, increase interferon-gamma (IFN-γ) production, and slower tumour growth [103]. However, when IL-33/ST2 signalling is chronically activated or dysregulated, it can trigger the mobilization of Tregs and M2-like macrophages, promoting a suppressive environment that facilitates immune escape [104]. In addition, activation of innate lymphoid cells group 2 (ILC2) and mast cells by IL-33 facilitates angiogenesis, fibrosis, and extracellular matrix remodelling, features that support tumour progression and metastasis [105].

The prognostic significance of IL-33/ST2 signalling activity in breast cancer remains an active area of investigation. Some studies have shown that elevated serum levels of soluble ST2 (sST2), a decoy receptor that neutralizes IL-33, are associated with advanced disease and poor prognosis [106]. Conversely, higher intratumoral expression of IL-33 may predict better response to chemotherapy or immune checkpoint inhibition therapy in some molecular subtypes of breast cancer [107,108]. In oestrogen receptor-positive breast cancer, elevated levels of IL-33 and sST2 are associated with tumour aggressiveness and resistance to treatment [109]. Furthermore, sST2 may serve as a marker of systemic inflammation and tumour burden, potentially useful in monitoring and stratifying patients [110].

In addition to TNBC, recent studies suggest that IL-33/ST2 signalling may play a role in other breast cancer subtypes. Circulating soluble ST2 (sST2) levels are elevated in ER-positive breast cancer and show dynamic perioperative changes, supporting its potential as a biomarker for disease monitoring and treatment response [111]. Transcriptomic profiling indicates increased IL-33 expression not only in TNBC but also in luminal B tumours [107]. Furthermore, stromal fibroblast-derived IL-33 has been shown to promote metastatic colonization in experimental breast cancer models by shaping a pro-metastatic immune microenvironment [112]. Collectively, these findings suggest that IL-33/ST2 signalling contributes to tumour progression across multiple breast cancer subtypes and may represent a therapeutic target beyond TNBC.

From a therapeutic perspective, manipulation of the IL-33/ST2 axis offers a dual opportunity. It can be inhibited to attenuate tumour-promoting inflammation, or it can be exploited to enhance the immune response in tumours with existing involvement of anti-tumour immunity. New therapeutic approaches, which are under preclinical evaluation, include the use of antibodies that neutralize IL-33 or ST2, especially in tumours with intensive accumulation of Treg cells or fibrous stroma [96,108,113]. Alternatively, selective enhancement of IL-33 signalling in effector immune cells, while avoiding stimulation of suppressive populations, may enhance antitumor immunity without affecting tolerance mechanisms [99]. Recent findings also support the integration of the use of IL-33 in combination therapy together with PD-1/PD-L1 inhibitors, chemotherapy, or radiotherapy, with the aim of exploiting its immunostimulatory properties while minimizing unwanted side effects [114].

The IL-33/ST2 signalling axis has multiple effects on breast cancer biology. It influences the composition and functional phenotype of tumour-infiltrating leukocytes, tumour aggressiveness, and therapeutic response in a manner that is largely dependent on the local microenvironment. A proper understanding of this duality is essential for the development of strategies that shift the balance towards effective antitumor immunity, paving the way for usage of IL-33/ST2 signalling axis as both a biomarker and a therapeutic target in personalized breast cancer treatment.

### Combined targeting of IL-33/ST2 and PD-1/PD-L1 signalling pathways: a novel synergistic approach to tumour control and immune activation

Recent experimental findings suggest that simultaneous manipulation of the IL-33/ST2 and PD-1/PD-L1 signalling axes represents a potent strategy for enhancing antitumor immunity and controlling tumour progression [108,115]. While the PD-1/PD-L1 axis is a well-established target in immune checkpoint therapy, the IL-33/ST2 signalling pathway has emerged as a novel potential modulator of anti-tumour immunity, with both pro- and anti-tumorigenic roles. The novelty of combined blockade or stimulation of these two pathways lies in their complementary and, in some cases, synergistic effects on immune system activation, tumour microenvironment remodelling, and the potential for therapeutic efficacy [108,115].

In mouse model of breast cancer, simultaneous blockade of both the IL-33/ST2 and PD-1/PD-L1 signalling pathways significantly delayed palpable tumour appearance (ST2^–/–^ + antiPD1 vs. WT= 15^th^ vs. 10^th^ day) and slowed tumour growth [108]. Co-blockade reduced primary tumour mass (ST2^–/–^ + antiPD1 vs. WT= 1,9 vs. 2,8 g) and volume (ST2^–/–^ + antiPD1 vs. WT= 450 vs. 900 mm^3^) by ∼50%, and prolonged survival of treated animals [108].

In a model of acute myeloid leukaemia (AML), it was shown that, although IL-33 administration alone can enhance CD8^+^ T-cell-mediated responses and prolong survival, the combination of IL-33 stimulation with PD-1 blockade induced even more potent antileukemic effects, including complete regression in a significant number of treated mice [115].

The dual modulation approach of the antitumor immune response led to a marked activation of key immune effector cells. In solid tumours, elevated expression of NK cell activating receptors NKp46 (ST2^–/–^ + antiPD1 vs. WT= 4,0 vs. 1,8%), NKG2D (ST2^–/–^ + antiPD1 vs. WT= 50 vs. 20%), FasL (ST2^–/–^ + antiPD1 vs. WT= 51,2 vs. 23,9%), increased IFN-γ production (ST2^–/–^ + antiPD1 vs. WT= 24,5 vs. 5,41%), and decreased expression of suppressive molecules such as IL-10 (ST2^–/–^ + antiPD1 vs. WT= 7,94 vs. 24,5%) were demonstrated [108]. These NK cells also showed enhanced survival, proliferation rate (Ki67 expression: ST2^–/–^ + antiPD1 vs. WT= 66,4 vs. 44,6%), and cytotoxicity, primarily *via* the perforin/granzyme B apoptotic pathway [108].

In parallel, T cells displayed an activated phenotype, with increased expression of FasL, CD69, and IFN-γ, and a shift away from regulatory T cells, as evidenced by decreased expression of FoxP3 (ST2^–/–^ + antiPD1 vs. WT= 0,83 vs. 3,07%) and IL-10 (ST2^–/–^ + antiPD1 vs. WT= 20,4 vs. 34,6%) [116]. Furthermore, increased accumulation and activation of NKT cells and M1 macrophages in the tumour microenvironment contributed to a potent proinflammatory and antitumor landscape [116].

Dual blockade was associated with epigenetic changes, increased expression of miRNA-150 (threefold increase in miRNAs expression compared to control) and miRNA-155 (double increase in miRNAs expression compared to control) in NK cells. These miRNAs are known to regulate NK cell activation status, IFN-γ production, and cytotoxic capacity, suggesting that epigenetic reprogramming plays a role in the formation of a potent antitumor response. In addition, increased activity of the transcription factors NFκB and STAT3 was observed in NK cells, further promoting their effector functions and survival within the tumour environment [108].

A key difference following dual blockade is the significant reduction in populations of immunosuppressive cells, including myeloid-derived suppressor cells (MDSCs) and regulatory T cells, both systemically (MDSCs: ST2^–/–^ + antiPD1 vs. WT= 53,0 vs. 73,6%; Tregs: ST2^–/–^ + antiPD1 vs. WT= 1,74 vs. 3,54%) and within tumours (MDSCs: ST2^–/–^ + antiPD1 vs. WT= 60,0 vs. 82,5%; Tregs: ST2^–/–^ + antiPD1 vs. WT= 0,83 vs. 3,07%) [108]. These changes are crucial for enabling sustained effector immune cell activity and overcoming the typical mechanisms of resistance and evasion seen in immunologically ‘cold’ tumours. Interestingly, while dendritic cells showed limited phenotypic changes in response to dual blockade, their role in antigen presentation may be complemented by enhanced effector cell function and reduced suppressive signalling, thereby shifting the immune balance towards tumour elimination [108].

Preclinical studies have shown that simultaneous blockade of IL-33/ST2 and PD-1/PD-L1 enhances antitumor immunity and prolonging survival in murine breast cancer models. In 4T1 and CT26 models, IL-33 pathway inhibition combined with anti-PD-L1 significantly suppressed tumour growth, reduced intratumoral Tregs, and extended survival. A bifunctional anti-PD-L1-sST2 fusion protein demonstrated superior tumour control compared with anti-PD-L1 alone [117]. For the clinical context, IL-33/ST2 axis therapies, including itepekimab (anti-IL-33), astegolimab (anti-ST2), and tozorakimab (anti-IL-33), have demonstrated favourable safety profiles in phase II–III trials for asthma and COPD, supporting feasibility for oncology testing (Table 1) [118–120]. These data support the feasibility of repurposing IL-33/ST2 blockade for oncology and combining it with PD-1/PD-L1 inhibitors in biomarker-selected cohorts.

**Table 1.** IL-33/ST2-Axis therapeutics currently in late-phase development for inflammatory airway disease.

Drug (target)	Mechanism of action	Current indication/setting	Development phase
Itepekimab (anti-IL-33 mAb)	Neutralizes IL-33, preventing ST2 receptor activation	Moderate-to-severe asthma (monotherapy or with dupilumab)	Phase II/III
Astegolimab (anti-ST2 mAb)	Blocks ST2 receptor, inhibiting IL-33 signaling	Severe asthma, COPD	Phase IIb (COPD), Phase III (asthma completed)
Tozorakimab (anti-IL-33 mAb)	Inhibits IL-33–mediated inflammatory responses	COPD, atopic dermatitis	Phase IIb/III

*Notes*: IL-33 – interleukin 33; ST2 – IL-33 receptor; COPD – chronic obstructive pulmonary disease.

**Figure 2.**
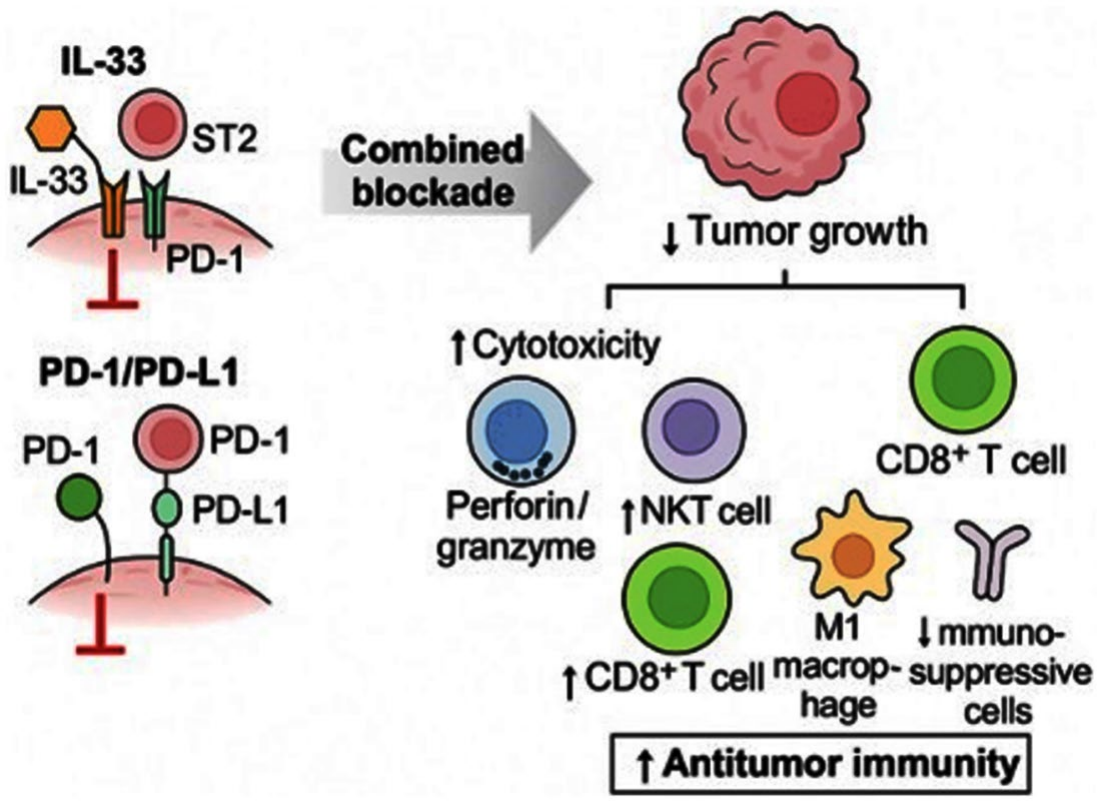
Combined targeting of IL-33/ST2 and PD-1/PD-L1 pathways enhances antitumor immunity and suppresses tumour growth. *Notes:* Schematic representation of the dual blockade of IL-33/ST2 and PD-1/PD-L1 signalling axes and their impact on the tumour-immune interplay. Inhibition of both pathways results in reduced tumour growth through multiple synergistic mechanisms: increased cytotoxic activity of NK and CD8^+^ T cells via perforin/granzyme pathways, accumulation of NKT cells and M1-polarized macrophages, and a marked reduction in immunosuppressive populations such as regulatory T cells and MDSCs. This strategy boosts both innate and adaptive antitumor immunity and offers a promising therapeutic approach for tumours resistant to monotherapies targeting individual pathways.

Simultaneous manipulation of the 2 signalling pathways IL-33/ST2 and PD-1/PD-L1, either through blockade in solid tumours or IL-33 stimulation in combination with PD-1 inhibition in leukaemia, achieves significant inhibition of tumour growth primarily through enhanced mobilization and activity of effector cells of innate (NK, NKT) and acquired (CD8^+^ T ly) immunity (Figure 2). These findings highlight the therapeutic potential of combined immunomodulation and support the idea of development of next-generation immunotherapies targeting multiple, intersecting immune regulatory pathways.

Moreover, multiple cohort and experimental studies have shown that elevated IL-33 expression is associated with poor prognosis in breast cancer. High levels of IL-33 expression correlate with shorter overall and disease-free survival, with hazard ratios approaching 1.5–1.8 in cohort analyses [121]. Enhanced IL-33/Wnt signalling facilitates the metastatic potential of the breast cancer, while genetic or pharmacological silencing of this axis inhibits tumour spread [122]. Together, these findings suggest that IL-33/ST2 overexpression defines a biologically aggressive subgroup and strengthen the rationale for therapeutic approaches that combine IL-33/ST2 inhibition with PD-1/PD-L1 blockade to improve patient outcomes.

### Predictive biomarkers and patient selection

An exploratory analysis from KEYNOTE-119 further nuances PD-L1 biology, suggesting complementary contributions of tumour-cell (TPS) and immune-cell PD-L1 to the CPS metric (CID = CPS − TPS). PMC.

Identification of reliable biomarkers is essential to optimize patient selection for dual PD-1/PD-L1 and IL-33/ST2 blockade. In metastatic TNBC, PD-L1 expression quantified by the combined positive score (CPS) 22C3 remains the only clinically validated biomarker, with a CPS ≥ 10 serving as an enrichment criterion in phase III TNBC trials [61]. Accordingly, we propose a PD-L1 CPS ≥ 10 as a pragmatic inclusion threshold for early dual-targeting studies. In addition to PD-L1, circulating soluble ST2 (sST2) represents a biologically plausible marker, reflecting IL-33 pathway activation and dynamically changing with disease status. However, its predictive utility for immunotherapy has not been prospectively validated and should currently be considered exploratory, ideally included as a stratification factor or a pre-specified variable in subgroup analyses [110].

Mechanistically, IL-33/ST2 signalling activates MyD88-dependent cascades leading to NF-κB and MAPK activation [72]. NF-κB is a known transcriptional regulator of CD274 (PD-L1) in multiple cancer models [123]. Therefore, chronic IL-33 signalling may upregulate tumour and stromal PD-L1 expression, promoting adaptive immune resistance. This provides a plausible mechanistic link by which IL-33/ST2 inhibition could downregulate PD-L1 and sensitize tumours to PD-1/PD-L1 blockade.

Methodological framework to establish combined PD-L1 CPS and sST2 cut-offs: we recommend starting with retrospective ROC analyses for each marker (Youden index), constructing a multivariable logistic/coxs proportional hazards model to derive a combined score, and validating *via* k-fold cross-validation. Prospective phase I/II studies should pre-specify these cut-offs and perform sensitivity analyses treating markers as continuous variables to avoid information loss.

In addition, pan-cancer bioinformatic analyses have identified AIMP2 as an immune-associated molecule whose aberrant expression correlates with tumour immune infiltration and patient survival, suggesting its potential as a biomarker in breast cancer immunomodulation [124]. Integrating markers such as AIMP2, PD-L1, and soluble ST2 may refine patient selection for dual-pathway immunotherapy. Additionally, miR-455-5p promotes triple-negative breast cancer growth and immune evasion by targeting SOCS3, a negative regulator of cytokine signalling [125] suggesting that microRNA-mediated modulation of ­cytokine pathways could influence responses to IL-33/ST2 and PD-1/PD-L1 co-inhibition.

### Clinical translation and therapeutic implications

Dissecting out the roles of the PD-1/PD-L1 and IL-33/ST2 signalling pathways in breast cancer biology has significant clinical outcome. Targeting the PD-1/PD-L1 axis has already shown significant clinical efficacy, particularly in triple-negative breast cancer (TNBC), where treatment with immune checkpoint inhibitors such as pembrolizumab and atezolizumab has significantly prolonged survival [60,61]. However, therapeutic responses are variable, highlighting the importance of identifying precise biomarkers and additional therapeutic targets to improve response [62,65].

The IL-33/ST2 axis represents an emerging therapeutic focus of interest. Therapeutic manipulation of this signalling pathway holds promise, either by suppressing its antitumor activities or by facilitating its immunostimulatory effects to enhance existing antitumor immunity [96,108]. Combination therapy involving modulation of the IL-33/ST2 signalling pathway with blockade of PD-1/PD-L1 may overcome resistance mechanisms to individual manipulation of these signalling pathways and consequently improve patient outcomes [108,113,114]. Clinical trials investigating these combination approaches are revealing novel synergistic effects, further emphasizing personalized treatment strategies adapted to individual tumour biology and immune response profile [108,115]. Several ongoing clinical trials provide important context for the dual-targeting strategy in breast cancer. Phase III trials are investigating PD-1/PD-L1 inhibitors in PD-L1-positive TNBC, demonstrating significant improvements in PFS and OS in populations with a CPS ≥ 10 [58,126]. In parallel, IL-33/ST2-targeting agents, including ipeceximab (anti-IL-33), astegolimab (anti-ST2), and tozoracimab (anti-IL-33), are in late-stage development for asthma and COPD, with favourable safety profiles [119,120,127] No oncology clinical trials of IL-33/ST2 blockade have yet been published. These findings motivate the design of early phase Ib/II studies combining IL-33/ST2 inhibitors with PD-1/PD-L1 blockade in biomarker-selected TNBC cohorts.

Although itepekimab/astegolimab/tozorakimab showed favourable safety in asthma/COPD cohorts, oncology patients differ in baseline inflammation, prior therapies and TME composition; consequently dose-finding oncology trials should start with conservative dose-escalation schemas and include PD markers (sST2, intratumoral PD-L1, TIL changes) and TME profiling. Potential unique toxicities to monitor include exacerbation or suppression of tumour-associated inflammation, unforeseen effects on tissue-resident innate cells, and potential additive immune-related adverse events when combined with PD-1/PD-L1 inhibitors. Taking all in account, we recommend baseline pulmonary assessment, serial cytokine panels, and early on-treatment biopsies as part of first-in-tumour studies

To date, IL-33/ST2 agents have robust safety data in respiratory disease trials (e.g. itepekimab, astegolimab), but oncology-specific phase I/II data are limited or unpublished. Consequently, we recommend cautious translation into oncology with detailed PD/TME biomarker work streams and conservative dose-escalation protocols.

### Resistance and combination strategies

Despite encouraging preclinical evidence, resistance to dual PD-1/PD-L1 and IL-33/ST2 blockade is likely to emerge. Mechanisms include compensatory upregulation of alternative inhibitory checkpoints, such as TIM-3, LAG-3 and TIGIT, persistence of immunosuppressive myeloid populations (MDSC, TAM) and loss of antigen presentation through alterations in the JAK/STAT pathway or β2-microglobulin mutations. Metabolic immune checkpoints, particularly adenosine signalling *via* CD73/CD39, may also limit T-cell activation in this context. Recent mechanistic studies show that combined blockade of PD-1 and TIGIT expands tumour-reactive CD8^+^ T-cell clones and restores effector function, supporting the rational design of triplet regimens (e.g. PD-1 + IL-33/ST2 + TIGIT) in biomarker-selected cohorts. Future studies should integrate genomic and immunological profiling to identify early signatures of resistance and guide sequential or combination approaches, thereby maximizing the lasting benefit of dual-axis immunotherapy [128].

Inhibition of IL-33/ST2 may reduce NF-κB mediated transcriptional upregulation of PD-L1 in tumour and stromal cells, thereby enhancing response to PD-1/PD-L1 blockade [129]. However, the resulting immunologic pressure may select for compensatory upregulation of alternative inhibitory receptors (e.g. TIM-3, TIGIT), arguing for the need for biomarker-guided monitoring and a possible sequential or triplet approach (PD-1 + IL-33/ST2 + anti-TIGIT/TIM-3) in clinical trials.

### Safety considerations and monitoring

Immune-related adverse events (irAEs) associated with PD-1/PD-L1 inhibitors are well characterized and include dermatitis, colitis, hepatitis, pneumonitis, and endocrine dysfunction (thyroiditis, hypophysitis), which can usually be treated with corticosteroids or immunosuppression [130,131]. In contrast, clinical trials of IL-33/ST2 axis therapies (itepeximab, astegolimab, tozoracimab) in asthma and COPD have reported predominantly mild to moderate events such as nasopharyngitis, upper respiratory tract infections, and headache, with low rates of serious treatment-related adverse events [118–120]. To date, no overlapping toxicities have been reported between these therapeutic classes, but the potential for additive immunomodulation warrants vigilance. For early-stage oncology trials, we suggest systematic monitoring, including baseline lung assessment, serial laboratory assessments (liver function tests, thyroid function), and rapid treatment algorithms for suspected irAEs [130,131].

### Future directions

Future research should aim to elucidate the dual roles of IL-33/ST2 signalling and further understand how these roles change between breast cancer subtypes and disease stages [80,94,96]. Advanced molecular techniques, including single-cell sequencing and spatial transcriptomics, can provide insights into cellular interactions and signalling dynamics within the tumour microenvironment [66]. Identification of patient-specific immune response profiles and mechanisms underlying resistance to existing therapies will be crucial for advancing precision oncology [62,63].

Furthermore, longitudinal studies assessing the prognostic significance of circulating and tissue-specific markers, such as soluble ST2 (sST2) and IL-33 expression levels, could improve patient stratification and follow-up [106,110]. Finally, preclinical models combined with bioinformatic approaches to identify predictive biomarkers of therapeutic outcome will enable the design of next-generation combination therapies [64,66]. Collectively, these efforts will contribute to the optimization of immunotherapeutic strategies and improve clinical outcomes in breast cancer patients.

## Data Availability

Data sharing is not applicable to this article as no new data were created or analysed in this study.
